# Continuity and change in human resources policies for health: lessons from Brazil

**DOI:** 10.1186/1478-4491-9-17

**Published:** 2011-07-05

**Authors:** James Buchan, Ines Fronteira, Gilles Dussault

**Affiliations:** 1 Queen Margaret University, Edinburgh, Scotland, UK; 2 International Health and Biostaitistics Unit, Institute of Hygiene and Tropical Medicine, Universidade Nova de Lisboa, Portugal

## Abstract

**Background:**

This paper reports on progress in implementing human resources for health (HRH) policies in Brazil, in the context of the implementation and expansion of the Unified Health System (Sistema Unico de Saúde - SUS).

The three main objectives were: i) to reconstruct the chronology of long term HRH change in Brazil, and to identify and discuss the precursors, drivers, and enablers for these changes over a long time period; (ii) to examine how change was achieved by describing facilitators and constraints, and how policies were adapted to deal with the latter; and (iii) to report on the current situation and draw policy implications.

**Methods:**

A mixed methods approach was used. A literature review was conducted using pre-defined keywords; and stakeholders were contacted and asked to provide relevant information, data and policy reports.

**Results:**

There are two key features of HRH change which are related to the implementation of SUS which merit attention: the achievement of staffing growth, and the improvement in HRH policy making and management. Staff growth rates across the period have been high enough to exceed population growth rates. As a consequence, the ratio of staff to population has improved. In 1990 the physician ratio per 1000 inhabitants was 1.12. In 2007, it was 1.74. Another critical factor in achieving staffing growth has been HRH policy making capacity and influence within the political establishment.

**Conclusions:**

Policies have had to adapt to changing circumstances, whilst focusing on sequential improvements aimed at achieving long term goals. The end objectives, of improving care and access to care, have been kept in view. No one Ministry could secure all the resources and impetus for change that has been required, hence the need for inter-ministry, inter-governmental and inter-agency collaboration, and the development of alliances of shared interest. Across the period of thirty years or more, not all initiatives have been equally successful, but a momentum has been maintained. There was no single long term plan or strategy, but in Brazil this has enabled the progress to be adapted and re-oriented as the broader context changed over the years.

## Background

### Introduction

This paper reports on progress in implementing human resources for health (HRH) policies in Brazil, in the context of the implementation and expansion of the Unified Health System (Sistema Unico de Saúde (SUS)).

Brazil has, over recent decades, sought to combine political will with a primary health care-oriented strategy and an improved capacity in health management and leadership, to build an integrated health services system (SUS). HRH development has played a determining role in this process, both as a strategy for scaling-up the health workforce to enable service delivery and to provide the capacity to implement the SUS vision and organization in more than 5000 municipalities country-wide.

There has been a long term policy commitment to the expansion of the SUS, which is based on primary/community care provision, with a focus on giving access to rural, remote and underserved populations, using community health workers and nurse technicians in a front line role, with support from qualified practitioners. As this process has occurred over a period across three decades, the approach can be seen to be an early example of policy interest and initiatives in what is now termed 'scaling up' the workforce and 'task shifting' to improve access to care [1].

The developments in Brazil therefore provide an opportunity to assess the policy implications, constraints and facilitators of the HRH aspects of achieving expanded coverage in a large federated country, through a focus on community health workers and primary care teams.

### Objectives

The main objectives of this paper are: (i) to reconstruct the chronology of long term HRH change in Brazil, and identify and discuss the precursors, drivers, and enablers for these changes over a long time period; (ii) to provide information on how change was achieved, by describing facilitators and constraints, and how policies were adapted to deal with the latter; and (iii) to report on the current situation and draw policy implications and lessons.

### Human resources for health policy in Brazil

Human resources for health policy implementation in Brazil has been conducted against a background of decentralisation and with a focus of municipality involvement. Brazil is a federal republic comprising of 26 states, a federal district, and more than 5000 municipalities. In 1988, the Constitution introduced the principle of universal access to health care, and that of the municipalisation of health services, thus initiating a complex process of decentralization [2].

The Brazilian health care system is segmented, with both private and public sources of financing [2]. In 2006, the annual national public healthcare expenditure as a proportion of GDP was 3.6%, with an additional 3.8% for private health) [3], giving a total of approximately 7.4% of total GDP on health. Three quarters (75%) of Brazilians use the public system exclusively [3].

The health system (SUS) provides free universal access to services, and is fully financed by public resources. It incorporated the health care network previously belonging to the Ministry of Health and the Instituto Nacional de Assistência Médica da Previdência Social (INAMPS). In addition, some large public enterprises, such as Petrobras or Banco do Brasil have created heath care plans of their own. These are considered to be part of the private health system. As such, they are regulated by the *Agência Nacional de Saúde *(ANS), and not by the *Secretaria de Atenção à Saúde *(SAS) of the Ministry of Health (MoH) [2]. The private system is voluntary; it includes numerous enterprise-based health plans financed by employees and employers. It also provides direct access to private providers by means of insurance and out-of-pocket payment [2].

From the start, Brazil has faced, and still faces, a range of HRH challenges which are familiar to any large country with a multi sector health care service. It has to achieve coverage across a large geography, with an unevenly distributed and growing population, coupled with the combined challenges of providing access to care in remote areas, and providing care in rapidly developing urban areas. Specific HRH challenges have included attracting and retaining health staff in remote and/or rural areas, tackling staff mal-distribution and over-specialisation, particularly in the physician workforce, retaining and motivating health workers, achieving consistent implementation of HRH policy with limited HRH management capacity, and optimizing the use of staff skills [2,4].

These main HRH challenges facing Brazil are not unique; they are present in many countries. However Brazil has developed specific approaches to addressing these challenges on a large scale. As such, there is wider interest and relevance to examining how the country has developed policies to meet these challenges. In particular, Brazil provides a long term case study on how to achieve significant growth in health staff numbers, which was achieved and sustained over more than 20 years, and an example of attempts to co-ordinate this action across different government departments and other stakeholders. Examination of what has been achieved, and how, is of relevance to the current focus on achieving "scaling up" of health workforce in many countries

## Methods

A mixed methods approach was used to generate the information necessary to complete the case study in Brazil. A literature review was conducted using pre-defined keywords (the search was conducted primarily in Portuguese, as little has been written in English on the Brazil HRH experience) to search specific databases (see Table 1). The names of important SUS stakeholders in the last 30 years were also used to search databases for published materials and other reports.

**Table 1:** Databases, keywords and stakeholders

Databases	
National Health Council	http://conselho.saude.gov.br/
Ministry of Health of Brazil	http://portal.saude.gov.br/saude/
Biblioteca Virtual em Saude	http://bases.bireme.br/cgi-bin/wxislind.exe/iah/online/?IsisScript=iah/iah.xis&base=LILACS&lang=i&form=F
LILACS	
National School of Public Health FIOCRUZ	http://www.fiocruz.br/bibensp/
Biblioteca de saúde pública	http://thesis.icict.fiocruz.br/php/index.php
Portal de teses e dissertações	http://www4.ensp.fiocruz.br/radis/
Revista RADIS	
SCIELO	
PubMed	

**Keywords**: sanitary reform (Reforma sanitária), unified health system (sistema único de saúde), human resources for health (recursos humanos de saúde), health professionals (profissionais de saúde), health workers (trabalhadores de saúde), policy (política), training (formação), education (educação), unification (unificação), development (desenvolvimento), Brazil (Brasil), municipalization (municipalização), (national health conferences) conferências nacionais

Source: authors

The literature review was complemented by interviews with stakeholders using a semi-structured questionnaire. These interviews provided additional reports and grey literature for review, as well as more specific detailed information on the process of reforms. The questionnaire was developed from information in the literature review and in consultation with Pan American Health Organization (PAHO) and the Secretariat of Labor and Education Management (*Secretaria de Gestão do Trabalho e da Educação na Saúde *- SGTES) of the Ministry of Health of Brazil. The main issues covered were HRH policies that enabled and/or supported the creation and development of SUS; critical HRH success factors for maintaining the SUS; main benefits and/or outcomes of HRH policies; major limitations and/or constraints to HRH policies; and views on how HRH policies linked to SUS have been adapted or changed over time.

Stakeholders were identified through a snowballing process based on dialogue with key officials and informants at the MoH and in the Brazil PAHO office, including those who had been involved throughout the period of reform. The objectives were to cover key policy makers, academics and researchers who had been involved closely in the process. Time and resource limitations meant that the focus was on key individuals and representatives of organisations. Eleven face-to-face individual interviews, one group interview (with four participants) and eight telephone interviews were conducted in September 2009 to provide the background information and specific details which were complemented by data and information from document review. Interviews were conducted by two members of the research team.

## Results

### Findings: The SUS and current HRH context in Brazil

This section provides a synthesis of findings drawn from the various information sources. There are two key features of HRH change which are related to the implementation of SUS which merit attention: the achievement of staffing growth, and the improvement in HRH policy making and management. Each of these is discussed in more detail below. This is followed by a more general presentation of the chronology of HRH change in Brazil.

### Staffing growth

Data analysis reveals that there has been a long term growth in the numbers of health workers employed in the SUS and in other areas of health care delivery. Recent estimates show that there are more than 2.5 million workers employed in the health sector; in terms of direct employment in formal skilled jobs this represents about 10% of the workforce [4].

In 2005, there were 715 137 doctors, nurses and dentists working in health care services (this represented an increase since 1999 of 22.8%, 35.8% and 100.7% more doctors, dentists and nurses, respectively). Of these, 52% were employed in the public sector, and of those in the public sector, two thirds (68%) were working for municipalities.

**Figure 1:**
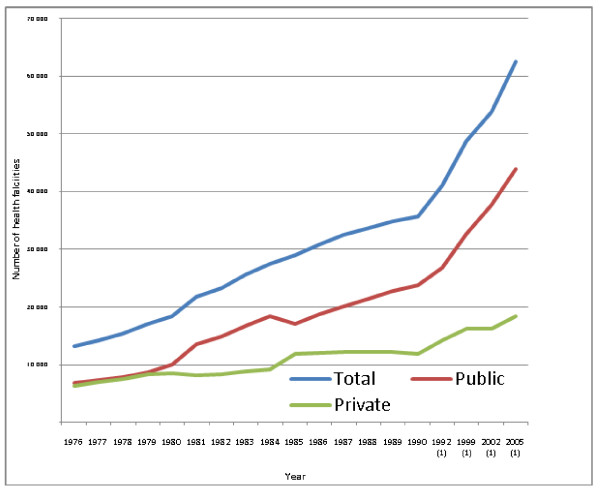
**Number of health facilities in Brazil since 1976 to 2005: total, private and public**. Note: (1) does not include diagnostic services Source: Instituto Brasileiro de Geografia e Estatística (IGBE)[18] Note: not sequential years

Staffing growth is related to a marked increase in the number of health facilities. The growth in the number of health facilities is shown in Figure 1. In 1980 there were approximately 18 500 health centres. By 2005 this had quadrupled to 62 500.

Results from interviews highlighted that in the mid 1980s there had been recognition of three main HRH constraints to the development of the SUS:

1. Insufficient skills of staff and limited access to training (50% of health workers had no qualifications, many with no formal skills), and maldistribution.

2. Low capacity to deal with local HRH management issues (raising the question, how do you change the system if local level HRH management capacity is low?).

3. An absence of linkage between the education and training sectors (universities) and the health services.

It was also recognized that the health system could not wait for the education system to prepare for new roles on its own initiative.

The solutions that were identified were:

1.

a. Expansion of technical training, up-skilling of public health personnel and auxiliary personnel (through the *Projeto de Profissionalização dos Trabalhadores da Área de Enfermagem *(PROFAE) and *Programa de Formação de Profissionais de Nível Médio para a Saúde *(PROFAPS) programs).

b. Use of the profile developed by Izabel dos Santos [5] - a shift in focus to "how to...", e.g. problem solving, and reflective thinking in training of health workers (this model already existed in technical schools for engineers).

2. Expansion of management capacity through programs such as Pólos Regionais de Educação Permanente em Saúde (PREPS) and Capacitação em Desenvolvimento de Recursos Humanos (CADHRU).

3. Use of funding mechanisms to stimulate change, e.g. providing incentives to promote curricular change in undergraduate courses, which are primarily a responsibility of the Ministry of Education (MoE), but have a shared program for curricular reform managed by the MoH.

Data analysis shows that staff growth rates across the period have been high enough to exceed population growth rates, and as a consequence the ratio of staff to population has improved. In 1990 the ratio of physicians per 1000 inhabitants was 1.12. In 2007, it was 1.74.

From 1990 to 2007, Brazil scaled-up the number of nurses and allied nursing professions but the most notable achievement of this scaling up process was at the end of this time period, in 2007. In that year--when compared to 2006--there was a reported exponential increase in the number of nurses, nurse technicians and nursing aides per 1000 inhabitants (from 0.24 to 0.94; 0.15 to 2.47; and 0.6 to 3.16, respectively) as a result of the deliberate policy of upgrading the nursing capacity linked to the PROFAE and PROFAPS policy initiatives. These intitatives are two key programs in relation to scaling up, and are examples of Brazil's efforts to expand HRH in terms of both number and qualifications (see Table 2 for a list of initiatives). This increase can be explained by the large number of technical schools that were involved, covering all regions of the country.

**Table 2:** Policies in relation to HRH in Brazil

POLICY	BEGINNING/END	DESCRIPTION
**Program Larga Escala**	80's	In service training program that aimed at qualifying middle and elementary cadres working in the public sector and that did not have access to formal training.

**CADHRU**	1987/...	Developed to aim at building HRH management capacity within SUS. It has had 3 phases: from 1987/1989 it was specially orientated to the train teachers, from 1992 to 2001 it became a speciallization course and now it is understood that it will contribute to the development and modernization of HRH institutional procesuss through capacity building.

**TELESSAUDE**	1999/...	Collaborative pilot project, between Federal Universities, private institutions and SUS; brought to 2700 family health teams and aiming at enhancing teams' ability to respond to primary care demands within SUS.

**PROFAE**	2002/2007	Aimed at expanding training of nurse technicians and nursing aides.

**PROMED**	2003/...	Aimed at financing curricular reform in medical schools directed towards the SUS

**PRO-SAUDE**	2005/...	Aimed at bridging the gap between HRH education and primary health care needs.

**PROGESUS *(****Programa de qualificação e estruturação da gestão do trabalho e da educação no SUS****)***	2006/...	Aimed at developing organizational guidelines and offering management tools, support and mechanisms for the modernization and professionalization of work management and education at municipal and state health secretariats.

**PROFAPS**	2007/2011	Based on a network of 319 technical schools; objective of training 735 435 health technicians by 2011.

**UNA-SUS (SUS Open University)**	2008/...	InterState network of collaborating academic institutions, health services and management services of SUS, to meet SUS' training and education needs; focus is on the use of distance learning, with free and shared access to learning materials.

**PET SAUDE**	2009/...	Aimed at integrating education, services and communities through in-service qualification and strengthening of primary health care professionals.

Source: authors

**Figure 2:**
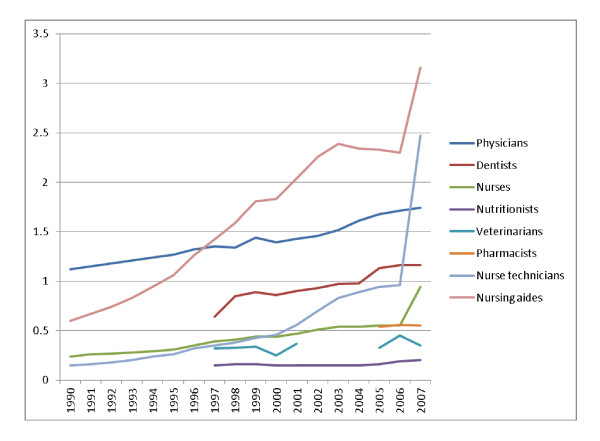
**Evolution of HRH ratios per 1000 inhabitants, from 1990 to 2007, per occupation**. Source: Instituto Brasileiro de Geografia e Estatística, (IGBE)[18]

One critical aspect of the progress of change in Brazil has been the emphasis on 'skilling up' as well as 'scaling up'. There has been a concerted attempt to increase the skills base of the main clinical providers of care, building on the pioneer work of Izabel dos Santos and others [5]. The emphasis has been on securing role development through mass training at technical schools and colleges throughout the country. This has been a major logistical challenge. PROFAE started in 2003 and was directed at expanding training of nurse technicians and nursing aides. Following the positive experience with PROFAE, PROFAPS was developed, based on a network of 319 technical schools spread all over the country. These have the objective of training 735 435 health technicians by 2011 that will then be hired to work within SUS (see Figure 2).

Whilst significant numerical growth has been achieved, there continue to be imbalances in the geographic distribution of HRH as illustrated in Table 3, as well as a lower ratio of nurses compared to doctors than in many other countries. For every physician in Rondônia, there are more than four in the federal capital district of Brasilia; for every nurse technician in Alagoas, there are more than fourteen in Mato Gross.

**Table 3:** HRH density (occupation per 1000 in habitants) per federal state in 1995 and in 2007

Federal State	Physicians		Dentists		Nurses		Nutritionists		Veterinarians		Pharmacists		Nurse technicians		Nursing aides	
	**1995**	**2007**	**1995**	**2007**	**1995**	**2007**	**1995**	**2007**	**1995**	**2007**	**1995**	**2007**	**1995**	**2007**	**1995**	**2007**

Rondônia	0.39	**0.81**	-	0.67	0.07	**0.45**	-	0.07	-	0.26	-	0.36	0.06	1.38	0.45	2.34

Acre	0.36	0.80	-	0.53	0.64	0.99	-	0.05	-	0.18	-	-	0.08	1.46	2.01	1.74

Amazonas	0.51	0.95	-	0.56	0.09	1.80	-	0.07	-	**0.06**	-	0.38	0.05	7.22	0.32	2.46

Roraima	0.27	1.15	-	0.65	0.15	0.94	-	0.09	-	0.22	-	-	0.16	1.85	1.08	3.87

Pará	0.52	0.77	-	0.42	0.08	0.55	-	0.10	-	0.16	-	0.28	0.03	2.23	0.44	1.62

Amapá	0.33	0.82	-	0.53	0.12	0.68	-	0.09	-	-	-	-	0.56	4.93	0.24	0.98

Tocantins	0.71	1.06	-	0.89	0.09	0.95	-	0.05	-	0.42	-	0.45	0.36	3.29	0.21	1.13

Maranhão	0.39	0.59	-	**0.33**	0.13	0.51	-	**0.03**	-	0.14	-	0.25	0.54	1.81	0.28	0.97

Piauí	0.5	0.84	-	0.56	0.19	0.76	-	0.14	-	0.20	-	0.16	0.21	1.62	0.47	2.02

Ceará	0.68	0.95	-	0.53	0.64	0.78	-	0.09	-	0.14	-	0.31	0.16	0.86	2.14	2.23

Rio Grande do Norte	0.85	1.21	-	0.78	0.07	0.75	-	0.20	-	0.13	-	0.58	0.06	1.45	0.6	3.10

Paraíba	0.85	1.17	-	0.79	0.48	1.10	-	0.14	-	0.16	-	0.51	0.16	1.89	0.87	2.16

Pernambuco	0.95	1.33	-	0.65	0.27	0.62	-	0.13	-	0.30	-	0.25	0.09	1.53	0.74	2.33

Alagoas	0.88	1.16	-	0.63	0.05	0.56	-	0.17	-	0.11	-	0.21	0.03	**0.87**	0.24	2.23

Sergipe	0.78	1.20	-	0.63	0.29	0.78	-	0.05	-	0.16	-	**0.20**	0.19	1.29	0.74	3.29

Bahia	0.72	1.02	-	0.53	0.12	0.61	-	0.12	-	0.15	-	0.23	0.03	3.00	0.38	3.39

Minas Gerais	1.19	1.71	-	1.36	0.29	0.75	-	0.12	-	0.34	-	0.59	0.29	2.18	0.97	3.10

Espírito Santo	1.31	1.81	-	1.16	0.18	0.81	-	0.12	-	0.19	-	0.67	0.32	3.47	0.49	1.66

Rio de Janeiro	2.87	3.37	-	1.64	0.41	1.21	-	0.41	-	0.42	-	0.53	0.85	3.79	2.13	4.46

São Paulo	1.71	2.28	-	1.75	0.39	1.05	-	0.27	-	0.43	-	0.70	0.16	1.16	1.27	4.34

Paraná	1.1	1.60	-	1.31	0.3	0.76	-	0.21	-	0.51	-	**0.98**	0.09	1.04	0.73	2.91

Santa Catarina	0.95	1.67	-	1.30	0.31	0.98	-	0.19	-	0.41	-	0.93	0.31	2.84	0.76	2.39

Rio Grande do Sul	1.61	2.08	-	1.17	0.45	1.25	-	0.35	-	0.62	-	0.76	0.24	4.87	1.97	2.86

Mato Grosso do Sul	0.93	1.45	-	1.22	0.02	0.66	-	0.15	-	**0.95**	-	0.68	0.03	1.83	0.26	2.64

Mato Grosso	0.57	1.12	-	1.00	0.16	**4.04**	-	0.15	-	0.58	-	0.62	0.05	**12.66**	0.28	**6.49**

Goiás	0.96	1.45	-	1.16	0.21	0.66	-	0.09	-	0.44	-	0.63	0.59	3.05	0.54	1.53

Distrito Federal	2.4	**3.57**	-	**2.18**	1.2	1.75	-	**0.47**	-	0.49	-	0.71	2.12	6.22	3.37	4.58

**Brazil**	**1.3**	**1.7**	-	**1.2**	**0.31**	**0.94**	-	**0.20**	-	**0.35**	-	**0.55**	**0.26**	2.47	1.06	3.16

Source: IDB, 2008 [3].

The percentage of health care professionals who work part-time is reported to be above 40% except for family health physicians, residents and clinical engineers, which might suggest that a significant proportion of these professionals has more than one job (see Table 4).

**Table 4:** Percentage of full-time and part-time work and relationship with employer per higher education health care professional in 2007

Occupation	Work			Relationship with employer		
	
	Full-time	Part-time	Not Known	Hired	Subcontracted	Others
	**14.9**	**45.0**	**40.0**	**39.3**	**40.6**	**40.6**

General Surgeon	**16.1**	**50.0**	**33.9**	**45.9**	**14.1**	**40.0**

General Practitioner	**21.1**	**62.7**	**16.2**	**65.4**	**12.8**	**21.7**

Geriatrist	**13.6**	**57.1**	**29.4**	**48.1**	**12.3**	**39.6**

Obstetrician-Gynecologist	**15.9**	**56.8**	**27.3**	**51.8**	**12.2**	**35.9**

Family Health Physician	**64.4**	**32.3**	**3.3**	**83.8**	**11.3**	**4.9**

Resident	**66.4**	**21.1**	**12.5**	**61.0**	**14.9**	**24.0**

Dentist	**29.1**	**64.7**	**6.2**	**79.1**	**7.4**	**13.5**

Pathologist	**31.3**	**46.3**	**22.4**	**65.5**	**10.6**	**23.9**

Pediatrician	**17.1**	**64.7**	**18.2**	**61.0**	**12.9**	**26.1**

Psychiatrist	**15.6**	**70.6**	**13.8**	**68.3**	**10.4**	**21.3**

Radiologist	**24.6**	**52.9**	**22.4**	**53.1**	**14.2**	**32.7**

Public health expert (Sanitarista)	**17.9**	**75.0**	**7.1**	**85.1**	**6.0**	**8.9**

Other medical specialties	**16.3**	**56.0**	**27.7**	**49.9**	**12.0**	**38.2**

Social assistant	**42.1**	**54.7**	**3.2**	**87.5**	**7.5**	**5.1**

Biochemist/Pharmacist	**45.0**	**48.7**	**6.3**	**83.8**	**6.2**	**10.0**

Nurse	**48.3**	**48.2**	**3.4**	**88.4**	**7.5**	**4.1**

Clinical Engineer	**69.1**	**17.3**	**13.6**	**80.8**	**9.6**	**9.6**

Medical Physicist	**31.6**	**52.2**	**16.2**	**57.9**	**17.0**	**25.1**

Physiotherapist	**27.5**	**57.4**	**15.1**	**63.0**	**11.9**	**25.0**

Speech therapist	**20.8**	**65.1**	**14.2**	**62.8**	**11.3**	**25.9**

Nutritionist	**38.2**	**55.1**	**6.7**	**77.8**	**9.1**	**13.0**

Psychologist	**23.7**	**66.9**	**9.4**	**71.9**	**8.9**	**19.2**

Other	**30.3**	**46.5**	**23.2**	**59.7**	**9.7**	**30.6**

Source: IBGE, 2007 [1].

Although the majority of health care professionals are directly hired by employers, a significant percentage is sub-contracted, such as anaesthetists (Table 4).

The available data highlights significant staffing growth across the last 20 years; however it has been uneven from one category to another, and unevenly distributed among regions.

Another method of assessing staffing is to compare Brazil with other countries. Such comparisons are fraught with difficulty--in part because there should be clear criteria for selecting country comparators--but more importantly because HRH data is often not comparable, being based on differing definitions, and often incomplete or out of date. This caveat should be in mind when reviewing the data in Table 5, which shows some comparisons drawn from the WHO World Health Statistics 2010. This should be taken only as a broad based illustration of the possibilities of comparison, and looks at two similar countries in South America, other countries at a similar ranking on the Word Bank table of level of development (Mexico, Malaysia and Turkey) and Canada.

**Table 5:** Country comparisons: Expenditure on health, and staff: population ratios, 2007

Country	Total expenditure on health as % of GDP	Per capita expenditure on health at average exchange rate (US $)	Physicians per 10 000 population	Pharmaceutical personnel per 10 000 population	Dentistry personnel per 10 000 population	Nursing and midwifery personnel per 10 000 population
Argentina	10	663	32	5	9	5

BRAZIL	8	606	17	6	12	29

Canada	10	4409	19	8	12	100

Chile	6	615	11	-	4	6

Malaysia	4	307	7	1	1	18

Mexico	6	564	29	8	14	40

Turkey	5	465	15	3	2	19

Source: WHO World Health Statistics 2010 [19]

The data in the table highlights that the HRH indicators for Brazil are not dissimilar to those in the other countries listed (other than Canada), but Brazil reports a higher ratio of "nursing and midwifery personnel" than the other countries, and a lower ratio population/physician than Mexico and Argentina.

The overall message is that the staffing growth was not the result of any one policy or initiative. A sequence of polices were enacted to create the conditions for staffing growth, as well as to provide the funding and training mechanisms which made the scaling up possible. Within a relatively decentralised system it was also clear that the process of decentralization gave more visibility to policy initiatives which otherwise would not be perceived to be 'real' at local level; there was therefore a process of learning and adaptability across the three main levels of government. Stimulus was provided to the training/education sector to ensure that ambitious targets for staffing growth could be met.

### HRH Management

One critical factor in achieving staffing growth in Brazil has been the HRH policy making capacity and influence within the political establishment. Since 2003, the policy making focus has been the Secretariat of Labor and Education Management in Health (SGTES) which was created as the MoH organ responsible for HRH issues in Brazil. SGTES is responsible for policies and strategic planning of HRH, namely training, education and regulation. The two additional main areas under development by SGTES are work management and education management.

**Figure 3:**
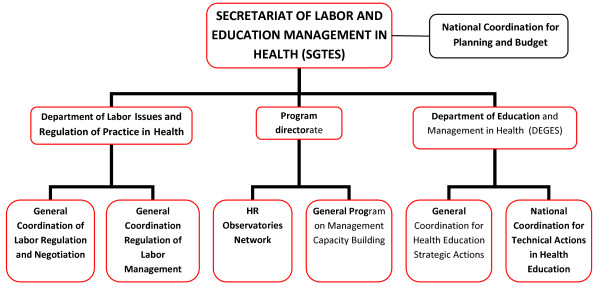
**Organigram of the MoH and Secretariat of Labor and Education Management in Health**. Source: adapted from [4]

In the first case, the emphasis is on workers' participation as a driver for SUS effectiveness and efficiency. SGTES main actions in this field have been:

(i) to improve the working conditions within the SUS (National Program for Precarious Working Conditions - *Desprecariza *SUS);

(ii) the regulation of HRH mobility (including internationally within Mercosul and Latin America);

(iii) the development of guidelines for planning and execution of the Work Management National Policy for SUS;

(iv) the professionalization of HRH management at State and Municipal level (PROGESUS);

(v) the regulation of work (careers, salaries), and

(vi) the development of a comprehensive HRH information system about the health labor market in Brazil

(see Figure 3 for SGTES structure).

In relation to the management of education, SGTES is only responsible for in-service training and education. Pre-service training is the responsibility of the MoE, but efforts are being made to converge both MoH and MoE interests in order to account for SUS HRH needs. This includes the range of programs highlighted in Table 2.

The improvements secured through HRH scaling up were built on foundations developed across a long time period. It is important to develop an understanding of the long time period and key milestones during this period that enabled and contributed to HRH change in Brazil

### The chronology of change

Table 6 traces the main chronology of the development of the HRH elements of SUS. This underlines that there were a series of precursors which helped prepare the ground for the implementation of SUS from the mid-1980s onwards, and illustrates that the policies used across the time period had to be adapted, refreshed and altered in order to maintain momentum and respond to changing political realities and priorities.

**Table 6:** Timeline of the development of the HRH elements of SUS

1920s	Policy to establish social security insurance (initially covering workers living with families and rural workers) that was finally established in 1966 with the creation of *Instituto Nacional de Previdência Social*.
**1960-1970s**	Social medicine departments created in the universities of São Paulo, Campinas, Ribeirão Preto, Minas Gerais and Rio de Janeiro. This led to development of *Movimento Sanitarista *which advocated and militated for universal access to care [9]. The development of this movement found fertile ground in the *Centro Nacional de Recursos Humanos do Instituto de Pesquisa Económica Aplicada *(CNRH/Ipea), in the financing agency *Financiadora de Estudos e Projectos *(Finep) and in the PREPS Program. In the late 1970s the Brazilian Association for Collective Health (ABRASCO) was created and there was the academic consolidation of the *Movimento Sanitarista, *with the development of a post-graduate course in collective health. This course bridged the gap between several academic institutions. It also set the basis for the latter discussions that occur in the National Health Conferences.

**1974 onwards**	Beginnings of focus on social determinants of health and of reform of medical curriculum: rural internship and need to provide HR in underserved areas

**1975**	PAHO/MoH initiates new teaching method: PREPS

**1976**	Beginnings of Governmental programs to extend health coverage to the rural and underserved population (PIASS)

**1977**	Creation of a mandatory rural internship for medical doctors in Minas Gerais

**1980**	Development of *Programa larga escala *(training of auxiliary and elementary personnel), based on a new pedagogic approach developed by Paulo Freire.

**1982 to 1986**	Development of PREV Saúde (the Brazilian health plan), with important HRH component.

**1985**	End of dictatorship - several key appointments in Ministry of Health; HR Secretary within MoH

**1986**	8^th ^National Health Conference - sets the basis for the *Sistema Único de Saúde (SUS)*, a health services system based on universal access, equality and equity and a decentralized model.

**1988**	The fundamental right to health, and the State's duty to account for citizens' health, are mentioned in the Brazil constitution of 1988. SUS is legally created and in 1990 SUS regulating laws are published

**c. 1991-1993**	Economic and financial crisis compromises implementation of SUS

**1996**	Legal norms and laws had been formulated; the SUS had begun to be implemented.

**2003**	SGTES established to handle HRH in a strategic way (National high level commission (Ministry of Health, Ministry of Education)

**Sept 2006**	Career guidelines approved by *Comissão Intergestores Tripartite *(CIT) and sent to the National Health Council

Source: authors

While most of the interventions specific to the HRH components of the SUS have occurred in the last 25 years, these precursor policies had set the scene, both for the implementation of SUS, and for the establishment of the HRH components. The ground had to be prepared in advance of the formal use of HRH policies, in terms of the establishment of the necessary linkages between health and education sectors, and of the long term overall coherence of policy direction.

One could consider starting point in the establishment of SUS to be as early as 1923, when one of the first health policies to create social security insurance was introduced, for certain categories of workers.. This coverage was extended during other governments. The principle of extending coverage to relatively underserved communities had been established. The full links between HRH development and education sector policy and change cannot be examined within this paper (see e.g. [6] and [7]) but it is evident that the role of the education sector, as training provider and as policy shaper, has been central to developments.

In terms of assessing where the roots of the HRH components of SUS first developed, several initiatives underway in the 1960s made significant contributions. Social medicine departments were created in universities in São Paulo, Campinas, Ribeirão Preto, Minas Gerais and Rio de Janeiro. The primary focus of some of these departments was on generating knowledge in this area, while others were dedicated to training with a social medicine perspective (Minas Gerais and Rio de Janeiro). These initiatives created the basis for the social determinants movement [8] and later the public health reform, which was influential, both in supporting the establishment of SUS and in ensuring that HRH elements were considered as central to that establishment.

Until that time the Ministry of Health focused mainly on combating endemic diseases, and health services were mainly provided by social security insurance. There were only 35 health units belonging to the Ministry of Health and there was little linkage or co-ordination with training institutions. The social determinants movement in medical schools created awareness that there was a need to integrate health care services.

From 1974, the influence of the social determinants of health model became more apparent, with the reform of the medical training curriculum: there was an increased emphasis on rural internships and the need to provide trained staff in underserved areas. In 1977, the first mandatory rural internship was created in Minas Gerais.

In 1976, PAHO, the MoH and the MoE initiated PPREPS, a program to promote the adequacy between HRH education and training to health services system demands such as universal, integrated, decentralized and progressive coverage, and population's expectations [9].

This was followed in 1981 by the introduction of the *"Programa Larga Escala" *which aimed at training basic and elementary health personnel, of whom 50% had no formal training, based on new pedagogical approaches, namely Piaget's genetic psychology, Joffré Dumazedier's adult training methodology and Paulo Freire's participatory methodology [10,11]

The period of 1982 to 1986 then saw the development of the program *Prev-Saúde, *the first health plan, which had a significant HRH component. The aim of the plan was to build a network of health centres and general hospitals. It is generally acknowledged that the plan failed because there were divergences between the ministries of health and social affairs in terms of priorities and approaches - but it did establish the foundation for the *Ações Integradas de Saúde, *and reflected an initial attempt to align the interest and work of the two ministries.

The year 1985 marked the end of the military regime, which had been in place since 1964, and several key appointees to the Ministry of Health at this time were part of an informal network that had been involved in previous activities to promote primary care and to improve services to the underserved. They were now in positions of power and influence within the health and education policy domains, and could move forward with the realization and implementation of these ideas. This included key senior staff appointments within the HRH Secretariat of the General Secretariat of the Ministry of Health.

During the 1980's, the Brazil office of PAHO also acted as a type of "think tank", providing protected space for some of these key planners to debate and work out their original ideas prior to implementation. These individuals had career trajectories which included working with PAHO, in government, and in universities at various times. This meant that the concepts regarding primary care-related reform were more fully formed when they entered public debate and consciousness, as they had already been tested and shaped in numerous debates. It also provided the basis for a future triumvirate of PAHO (PPREPS), Ministry of Health, and Ministry of Social Affairs to act as a coalition of shared interest, using a more collaborative approach.

In essence, the implementation of SUS and the establishment of a state-based on democratic principles were interdependent--the introduction of democracy was an enabler of SUS, whilst the establishment of SUS itself was a part of the process of achieving and sustaining the democratic process.

Another major milestone was in 1986, with the 8^th ^National Health Conference, which set the stage for the introduction of the SUS, a delivery system based on universal access, equality and equity. In essence it was the operationalization of the social determinants of health vision [2]. The first National Health Conference had taken place in 1941 and aimed at debating the sanitary situation and health service delivery in Brazilian states. Since then there have been thirteen Health Conferences (the last one being in 2008). The National Health Conferences are events where the developments and problems of SUS are discussed and health policy reformulation proposed. The attendees are stakeholders coming from a range of sectors of Brazilian society. National Health Conferences are preceded by State and Municipal Conferences that happen all over the country. The theme of these conferences is the same and they work as a think tank for the National Health Conference [12].

The realities of the establishment of SUS were difficult initially because of different views about how SUS should be structured and implemented. Some stakeholders advocated that the SUS should be a system where the State would be present at every level as a provider and regulator; but public services at the time did not have the capacity to play such roles (e.g. 75% of hospital beds were private, most of them in the not-for-profit network of *Santas Casas da Misericordia*).

Some key participants in this debate were both educators and working in HRH. The creation of SUS was therefore not a paper-based strategy isolated from the realities of HRH. Those involved had a vision for the future which was tempered by an appreciation of the practical realities of implementing strategy. They were thinking and debating the key aspects of the strategy but were also thinking about the HRH policies and issues necessary to make it happen.

In 1988, the new Constitution of Brazil established the legal base of SUS (Articles 196-2000) - "an important set of social rights, health as a duty of the State and a right of the population". In the early 1990s (c1991-1993), the image of what the SUS should be like became clearer, but the economic and financial crisis that Brazil was facing at the time did compromise its implementation. However, by 1996 legal norms and laws had been formulated and implementation accelerated. It has continued to the present day with an additional critical moment of development occurring in 2003, when SGTES was set up to strategically manage and plan HRH, focusing on education and working conditions.

## Discussion

The implementation of the HRH elements of SUS in Brazil has been based on various key pillars/concepts, which have evolved over time whilst retaining some core principles, and which are now closely linked to identifiable functions or departments within the Ministry of Health:

• scaling up the stock of auxiliaries by means of a new curriculum- e.g. scaling up of skills, not just numbers (there had been a recognition from the beginning that there was a need for a critical mass to achieve results, and secure broader support for SUS at an early stage): PROFAE, PROFAPS, *Programa Larga Escala.*

• re-orientation of education strategies to link curricula more closely to priority needs of society--e.g. family health, primary care--and move away from overspecialization (PROSAÚDE),

• emphasis on lifelong learning, in-service training to keep skills updated (PETSAUDE),

• focus on multi-professional teamwork; training in management

○ PROGESUS - Program for Improving the Qualification and the Mechanisms to Manage the Workforce and the Education within the National Health System (SUS);

○ CADHRU (Training and development of manpower in Health);

○ UNASUS (National Health System's Open University).

• development of a network of HRH Observatories: analytical and policy formulation; testing; aiming to bridge the gap between SUS and academics.

• emphasis on 'co-operation' with professional associations and trade unions through the use of the so called 'Negotiation Tables'- similar to the tri-partite model promoted by the International Labour Organisation (ILO).

**Figure 4:**
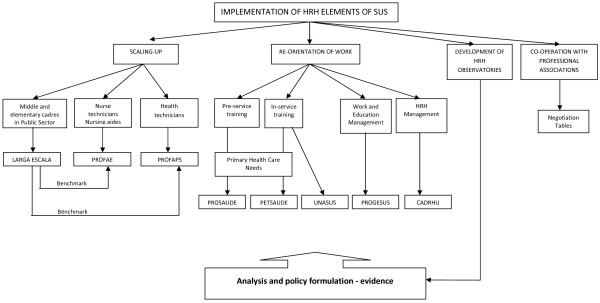
**Schematic representation of the implementation of HRH elements of SUS**. Source: authors

Figure 4 provides a schema of these main areas of action, and linkages between them.

The limited number of published evaluations of change in HRH issues in Brazil suggest that there has been significant progress (see, e.g. [13]), but that big challenges remain, such as ensuring that 'front line' health workers have career structures and job stability [14]. Broader based research on HRH elements of health sector reform reinforce the need to understand that HRH is only one component of change, and that sustained improvements cannot be achieved without a co-ordinated approach to HRH policy change, along with a recognition that there are no 'magic bullet' solutions. HRH policies need to be 'bundled' - they must be linked, developed and adapted over time for real impact [15]. In particular, the evidence base on role development and skill mix changes highlights that this is a process that requires a long term strategy linked to organizational change, regulatory change and supportive educational policies[16].

Overall, in Brazil there has not been a single detailed long term 'plan' or strategy for HRH change, but there has been a supportive vision. Some countries would regard this as a weakness; but in Brazil, it has enabled the progress to be adapted and re-oriented as the broader political, policy, and economic contexts changed over the years. There was no initial, detailed and fully formed 'blueprint'; such a thing would have been unlikely to survive political change across the time period. The broader, long term objectives and principles have not been challenged by key stakeholders and have remained at the core of the process of change, but the strategies developed and deployed to achieve and sustain change have been altered and revised across time, to maintain momentum and deal with emerging challenges and barriers. The primary focus has been on how to adapt, and if necessary, change the detailed content of pillars to keep progressing in the right direction. Targets for overall staffing growth have been used to focus policy actions and keep stakeholders engaged.

Within SUS, there continue to be difficult and challenging HRH issues [13,14]. At present, these include securing support from professional associations and trade unions for more flexibility, meeting the need to attract and retain staff in underserved, remote areas, and trying to reduce overspecialization. There are also legal constraints on allocation of funding to pay levels and rates. Other ongoing HRH challenges include the rigidity of local 'hiring and firing' practices, which limit organisational flexibility; the 'professional migration' of workers from, e.g., paediatric to primary care because the latter is better paid; and the need to improve the management of staff absenteeism and of dual employment.

There is also a need for better alignment of education and service needs--including accreditation and adaption of training courses. It is recognised that, in part, the latter will require increased emphasis on the use of newer technology to support the delivery of training packages and lifelong learning, with an increasing use of tele-health and e-health. As with most health services systems, there is also a need for better analysis, and more scope to network effectively in order to influence good practice, with the priority being to sustain a focus on front line delivery at the municipality level, supported by the state level.

### Limitations

The study could cover only some of the key individuals who were informants on HRH issues during the process of change in Brazil, and they were reflecting on past change, rather than contemporaneous developments. The focus primarily on lusophone literature should have provided access to the main published sources, but many aspects of policy change are contained in grey literature and unpublished sources, which are difficult to identify and access. A more detailed examination of broader issues related to social change and to education policy developments in Brazil would be of relevance.

## Conclusions

The policy message is clear: to secure sustained HRH change across a long period of time in a large country such as Brazil, policies have had to adapt to changing circumstances, whilst focusing on sequential improvements aimed at achieving long term goals. The end objectives--of improving care and access to care--have been kept in view. No one Ministry could secure all the resources and impetus for change that was required, hence the need for inter-Ministry, inter-governmental and inter-agency collaboration, and the development of alliances of shared interest. Across this long time period, and with significant shifts in the political process in Brazil, not all initiatives have been equally successful, but a momentum has been maintained.

A recent OECD broad-based review of human resource management at federal level in the government of Brazil noted that "The Brazilian public sector has played a crucial role in promoting stability and setting up the conditions for economic and social development" [17]. In this context there are broader issues of the performance of SUS to be considered, which link to the overall level of funding for health, and therefore for SUS, and for staffing of SUS. This relates in part to the growth of a relatively prosperous middle class in Brazil, and their reported preference to use private insurance, which could undermine the principles of SUS. If more Brazilians chose the private sector alternative, coverage and funding for SUS could then be more vulnerable, as fewer voices will call for its defence.

This highlights the current critical policy question for Brazil: how should SUS be re-oriented to meet this changing socio-demographic profile and priorities? Answering this fundamental policy question will require an appreciation that HRH has been at the core of health policy determination in Brazil in recent decades, and has been a significant enabler of change. Any new policy direction will have to take account of the HRH dimension.

## Competing interests

The authors declare that they have no competing interests.

## Authors' contributions

JB contributed to study design, fieldwork, analysis, report writing and final edit. IF contributed to study design, fieldwork, literature review, analysis, report writing. GD contributed to study design, literature review, analysis and report writing. All authors read and approved the final manuscript.
